# EHMTI-0206. Nigerian plants that are used for treatment of headache and migraine

**DOI:** 10.1186/1129-2377-15-S1-G33

**Published:** 2014-09-18

**Authors:** SA Saganuwan

**Affiliations:** 1 Veterinary Physiology Pharmacology And Biochemistry College Of Veterinary Medicine, University of Agriculture Makurdi, Makurdi, Nigeria

## Introduction

Migraine and headache are life-threatening ill-conditions that affect the active, industrious class, which may likely reduce productivity.

## Aim

Because plants serve as source of 1 in every 4 drugs on drug market, there is need for survey of literatures with a view to identifying Nigerian medicinal plants with potential activity against migraine and headache.

## Method

Literature survey and photography of medicinal plants that are used in Nigeria was carried out with a view to identify medicinal plants with therapeutic potentials against headache and migraine.

## Result

Out of 184 medicinal plants identified, 23 have activity against migraine 29 have activity against both headache and migraine and 132 have activity against headache respectively. Orchis mascula, Hollarhena floribunda, Malaleuca leucodeudron, Cinnamomum zeylanicum, Citrus aurantifolium, Luffa accutangula Brassica juncea, Brassica nigra, Ocimum basilicum, Citrus aurantium Petasites hybridus, Rothmania whitfeildi, Palisota hirsuta, Crassocephalum crepidioides, Pridelia micrantha, Parkia bicolor, Cycodiscus gabonensis, Abelmoschus maschattus, Adenia venenata, Culcasia scandens, Dioscorea dumentorun, Galinsoga parviflora, Kolobopetalun auriculatum, Matricaria recutita, Petveria alliance, Piper nigran, Spilanthes filicaulis, Tragia benthani and Nigella sativa have activity against migraine and headache. Decoctions, infusions, concoctions and vapour of roots, leaves, stembark, flowers, and oil of the plants are used for the relief of the conditions. Since hypertension can cause headache plants such as Nigella sativa, Ocimum basilicum, chloriandrum sativum may be used to treat both migraine and headache caused by hypertension.

## Conclusion

Therefore medicinal plant resources from Africa should be harnessed with a view to discovering additional novel drugs with activity against headache and migraine.

No conflict of interest.

